# Imaging Diagnosis of Right Ventricle Involvement in Chagas Cardiomyopathy

**DOI:** 10.1155/2017/3820191

**Published:** 2017-08-27

**Authors:** Minna M. D. Romano, Henrique T. Moreira, André Schmidt, Benedito Carlos Maciel, José Antônio Marin-Neto

**Affiliations:** Medical School of Ribeirão Preto, University of São Paulo, Ribeirão Preto, SP, Brazil

## Abstract

Right ventricle (RV) is considered a neglected chamber in cardiology and knowledge about its role in cardiac function was mostly focused on ventricular interdependence. However, progress on the understanding of myocardium diseases primarily involving the RV led to a better comprehension of its role in health and disease. In Chagas disease (CD), there is direct evidence from both basic and clinical research of profound structural RV abnormalities. However, clinical detection of these abnormalities is hindered by technical limitations of imaging diagnostic tools. Echocardiography has been a widespread and low-cost option for the study of patients with CD but, when applied to the RV assessment, faces difficulties such as the absence of a geometrical shape to represent this cavity. More recently, the technique has evolved to a focused guided RV imaging and myocardial deformation analysis. Also, cardiac magnetic resonance (CMR) has been introduced as a gold standard method to evaluate RV cavity volumes. CMR advantages include precise quantitative analyses of both LV and RV volumes and its ability to perform myocardium tissue characterization to identify areas of scar and edema. Evolution of these cardiac diagnostic techniques opened a new path to explore the pathophysiology of RV dysfunction in CD.

## 1. Introduction

Historically, physiology concepts about heart function considered the right ventricle as a kind of almost perfunctory chamber, although knowledge about ventricle interdependence was well recognized decades ago [1]. Weber et al., in an outstanding publication of 1981 in this field, postulated the following:

However, because the right and left ventricles are aligned in series and mechanically coupled, a perturbation in the mechanical events of one ventricle will influence the behavior of the other ventricle, and in nonsteady state conditions the output of the two ventricles may not be balanced. Eventually, however, a steady state of balanced outputs must occur if pulmonary or systemic venous congestion is to be avoided. [1]

Diastolic interplay between ventricles is easy readily understandable, mainly considering the function of pericardium as the common covering layer for both ventricles. However, although the presence of pericardium accentuates this interaction, animal experimental models with the pericardium excised showed that the diastolic interplay was still present [2]. There is also a systolic interplay, so that, during systole, the pressure in one ventricle will influence the systolic pressure developed in the other. The systolic interplay has been primarily demonstrated as the predominant influence the left ventricle exerts upon the right ventricle. This is because the opposite effect of lower systolic pressure of right ventricle influencing left ventricle performance is usually minimized under normal physiologic conditions. As the interventricular septum plays an important role in the interventricular pressure relationship, its function was assessed in classic animal experiments with total destruction of RV free wall but with maintenance of septum presence [3] and, in contrast, in experimental models of septum dysfunction because of infarction [4].

Based on these physiologic aspects and adding to the fact that many of the studies were designed on a volume-pressure relation basis, it is possible to understand why cardiology science neglected the RV as a “secondary chamber” or a “passing chamber” [5]. Nonetheless, knowledge about primary myocardial conditions affecting the RV, without concomitant pressure overload of this chamber, as in Uhl's anomaly and RV dysplasia, reinforces the need of other theories to explain the real influence of right ventricular function in cardiovascular physiology. The fact that RV dysplasia includes a phase of right-sided cardiac failure beyond the arrhythmic clinical complications gives an insight to the fact that advanced RV muscle impairment can be clinically significant and directly leads to intrinsic heart failure syndrome [6].

There is evidence that CD causes a peculiar right ventricle involvement first because evolution of the disease to heart failure usually includes prominent right heart failure symptoms in absence of or with only mild pulmonary congestion [7, 8]; second, because there is direct evidence of myocardial damage of the right ventricle in human myocardial biopsies and postmortem studies, as well as in animal experimental models of* T. cruzi* infection; and, finally, because there is a proportion of patients with early and isolated right ventricle dysfunction when evaluated by different cardiac image methods [9, 10].

However, exploring the right ventricle with imaging diagnostic tools is still a challenge. The accumulated evidence about ventricular systolic function in most diseases is derived from echocardiography. Two-dimensional echocardiography needs geometrical assumptions to estimate ventricular volumes and the right ventricle simply does not have a mathematically defined geometrical form.

This review explores the right ventricle anatomic and functional involvement in Chagas cardiomyopathy (CC) while highlighting the contribution of cardiac imaging diagnostic methods in the field.

## 2. Chagas Cardiomyopathy (CC)

CD was recognized by WHO as one of the world's 13 most neglected tropical diseases with a high prevalence of individuals at risk of infection in South and Central America and with the highest rates of mortality between all neglected diseases [14]. Although its prevalence had slightly declined through the last decade, it can reach 11.4% of heart failure causes in specific endemic regions of Brazil [15]. Although originally confined to poor rural areas of Latin America, migration movements contributed to spread CD to nonendemic countries in Europe, Canada, and USA [16, 17].

**Figure 1:**
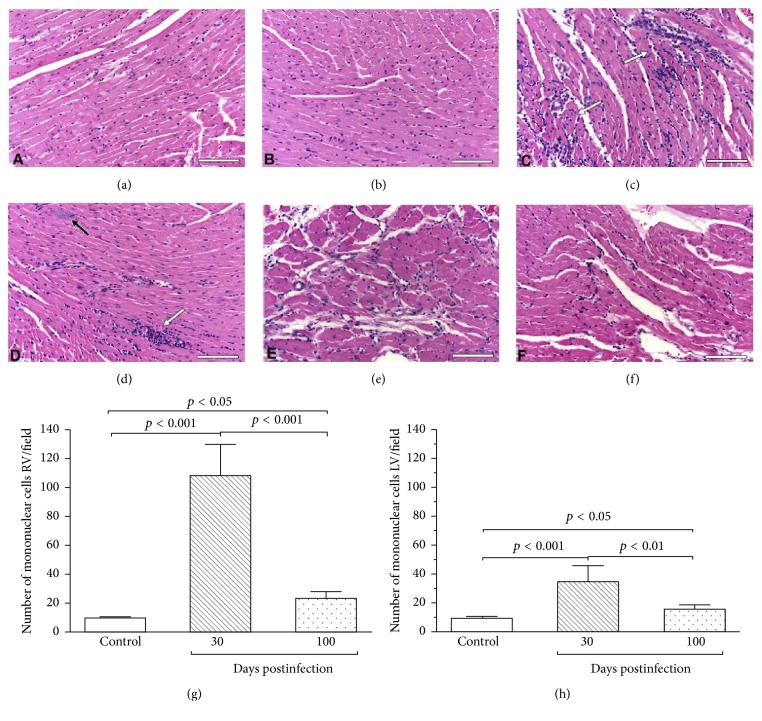
Histological analysis (HE) depicting normal cardiac fibers with regular interstitial space in the right (a) and left (b) ventricles. In a* T. cruzi*-infected mice, with 30 days post-Chagas infection (acute phase), there is an intense and diffuse myocarditis characterized by lymphomononuclear interstitial infiltrate (white arrow), multiple ruptured or unruptured pseudocysts (black arrow), and enlargement of the interstitial space ((c) and (d)). After 100 days of infection (chronic phase), the number of the lymphomononuclear inflammatory cells became significantly reduced and no parasites are detected ((e) and (f)). In this study, the numbers of interstitial mononuclear cells were quantified in both right and left ventricles. The number of cells was markedly increased at 30 days of infection as compared to 100 days of infection, mostly in the right ventricle ((g) and (h)). Bars = 100 *μ*m, *n* = 6/day/group. HE = Hematoxylin-Eosin stain. Adapted from [11]. Adapted with permission from Elsevier, license number 4120251034702.

CD is caused by the* T. cruzi* protozoan infection that leads, in most cases, to a myocardial chronic inflammatory response [7]. Myocardial damage is probably a result of imbalance between parasite persistence and adverse immune-inflammatory response [18] which result in a broad spectrum of tissue lesions. Myocardium disease is characterized by a low-intensity, slowly progressive but incessant myocarditis which leads to impairment of contractile function and dilatation of cardiac chambers [7]. Histologically, there is a widespread destruction of myocardial cells, edema, diffuse fibrosis, mononuclear cell infiltration, and scarring of the conduction system and the contractile myocardium [11, 19] (Figure 1). Early stages of the disease are still not fully understood, but conduction system abnormalities can occur before myocardium contractile function impairment [20]. Cardiac autonomic dysfunction may also be an early consequence of the loss of cardiac neuronal parasympathetic activity [21–23]. Microvascular coronary dysfunction also occurs and its mechanism is still not well known but is likely to contribute to contractile function damage [24–26]. Progression of the myocardium damage results in a scenario of widespread substitution by fibrous tissue, which is the core of both arrhythmogenesis and heart failure.

CD includes an acute and a chronic phase. Acute infection is usually underdiagnosed. There is a recent increase of cases secondary to reactivation from the chronic phase, by blood transfusion or transplantation of solid organs, and congenital or oral contaminations [27, 28]. Most of acute cases run through an asymptomatic clinical course. When symptoms occur they include fever, malaise, enlargement of liver, spleen and lymph nodes, and subcutaneous localized edema [7]. The grade and type of immunological response have a key role in controlling parasitemia during acute phase and permit most of patients to undergo to the chronic clinically silent indeterminate form of disease. However, some 30% of infected subjects will present symptoms and or signs of cardiac damage 10–30 years later [7]. Bradycardia and conduction system abnormalities can occur early in the evolution of disease, as well as frequent ventricular ectopic beats [17, 19]. Ventricular tachycardia is ominous and can be responsible for sudden cardiac death even in less advanced stages of the disease [29]. Other typical ECG alterations include right bundle branch block and left anterior fascicular bundle block, which can be combined [19, 30].

Myocardial damage typically causes regional contraction disturbances in the left ventricle and the virtually pathognomonic apical aneurisms [22, 31]. Global LV dilatation and systolic dysfunction are also often seen as the disease progresses. When heart failure supervenes, it is usually with biventricular manifestations and systemic congestion can be more impressive when compared to pulmonary congestion. Patients with cardiac form of CD are seen frequently in cardiac units and clinics all around South and Central America. They usually present with heart failure symptoms during recurrent hospital admissions. In many cases, symptoms and signs of systemic congestion are very prominent. They usually complain of lower limbs edema, weight gain, increase in abdominal volume, and pain associated with hepatomegaly. This conspicuous form of right-sided heart failure presentation was well described since the first reports on clinical CD [32–34] and still puts a challenge to treatment. Patients need high doses of diuretics and vasodilators and sometimes the use of intravenous inotropes, although it, with no proven benefit on mortality, is warranted as a symptomatic treatment resource [33].

Because of these peculiar aspects of CD cardiomyopathy, diagnostic tools capable of detecting early myocardial involvement in both ventricles can represent a first step towards an early and effective treatment of these patients before the development of more advanced fibrotic and irreversible changes.

## 3. Evidence of Right Ventricle Involvement in Chagas Disease (CD)

**Figure 2:**
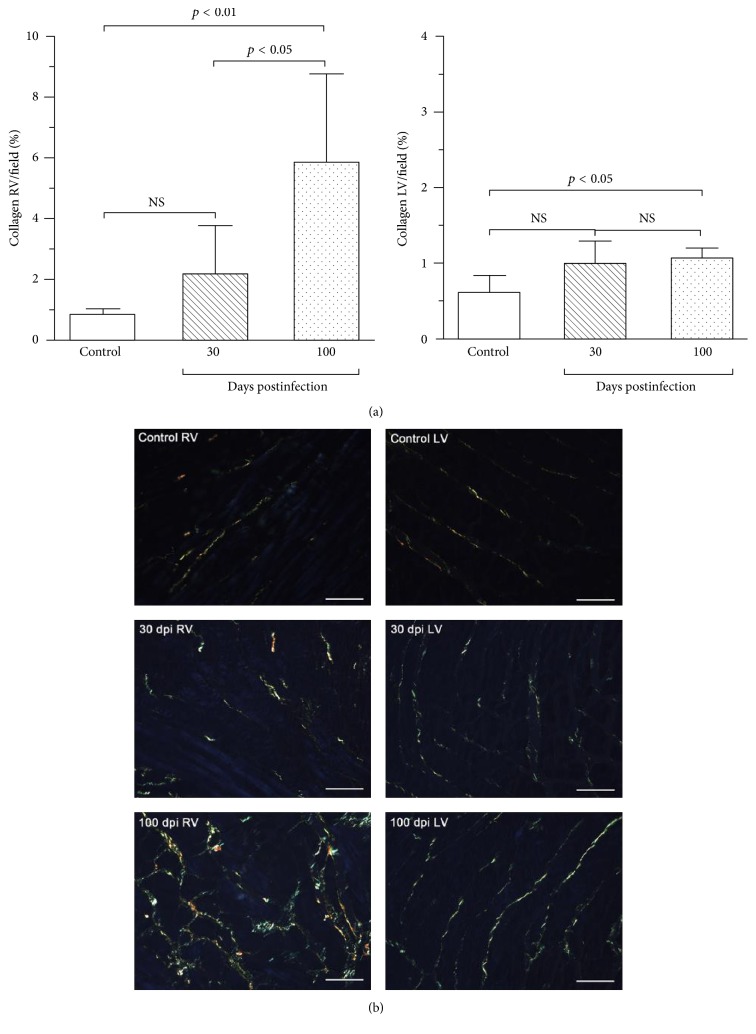
Interstitial collagen (Picrosirius red-stained sections) in controls and* T. cruzi*-infected mice. (a) The bar graphs show the mean fraction of fibrosis (%) in both right (left graph) and left ventricles (right graph). There is a tendency towards increased amount of collagen in acute phase (30 dpi) in both ventricles. The mean amount of collagen is significantly increased in the RV (600% higher) and LV (62% higher) in chronic phase (100 dpi). (b) Representative images illustrate these results clearly showing that the collagen increase was mainly perimysial. Bars = 50 *μ*m, *n* = 6/day/group. Adapted from [11]. Adapted with permission from Elsevier, license number 4120251034702.

**Figure 3:**
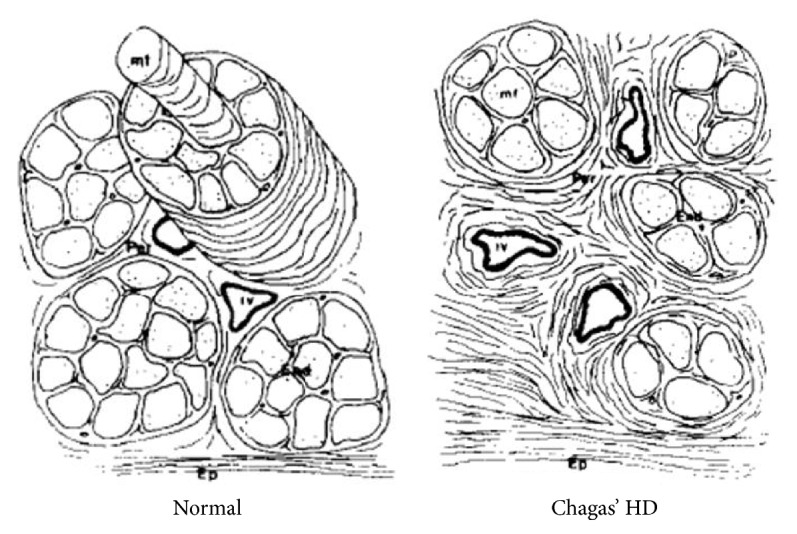
Schematic representation of the myocardial fibrosis patterns in control myocardium (normal) and in chronic Chagas' heart disease (Chagas' HD). From [12]. Adapted with permission from Elsevier, license number 4120260350349.

There is considerable evidence showing CC to be quite different from other forms of dilated cardiomyopathy from the pathologic standpoint [35]. Not only is there a prominent myocardial cell destruction associated with focally diffuse mononuclear cell infiltration, but also an intense interstitial fibrosis is seen (Figure 2), surrounding muscle cells and vessels (Figure 3) [12]. The process of inflammation and fibrosis is widespread and involves both the right [36] and left ventricles and has particular predilection for the cardiac conduction system and the apex of left ventricle [35, 37]. Higuchi et al., in a previous study using RV endomyocardium biopsies, compared ultrastructural changes in CD patients with different forms of the disease (indeterminate versus cardiac with and without ECG alterations) and did not encounter important differences among them. Since the pathological materials for these studies were obtained from the RV myocardial tissue and the ultrastructural alterations detected were similarly distributed in patients with the different forms of CD, these findings suggest an early involvement of the right ventricle ultrastructure in many patients [38]. Other studies with right ventricle biopsies also showed that myocardium lesions may be caused by a continuous process along all forms of the chronic disease and are found even in early stages of the disease (e.g., in patients with the indeterminate form of the disease) [39].

The peculiar clinical aspects of Chagas cardiac failure drove initial research to investigate the real role of right ventricle dysfunction in the disease physiopathology. In fact, previous studies reported on patients with CD showed that isolated right ventricle disturbances are frequent occurrences [21, 34]. It has also been reported that heart failure mortality in CD is higher when compared to mortality associated with heart failure due to ischemic causes or other cardiomyopathies [40, 41]. Among other possible factors, RV dysfunction prevalence may be responsible for high mortality of heart failure in chronic CD once; as in other causes of heart failure, it has independent prognostic impact [42]. It is also noteworthy that, in cases of reactivation of* T. cruzi* infection in immune suppressed patients, a remarkable degree of RV dilation and systolic dysfunction has been described [43–45].

Despite these findings, some controversy occurs in the literature regarding the detection of clinically apparent early right ventricle involvement in CC. Carrasco et al. [46] studied 60 subjects using cardiac catheterization and radiological contrast angiography, for both RV and LV, in patients with different forms of chronic CD and did not find right ventricular dysfunction or dilatation in a subgroup (1) of 14 patients with normal ECG and normal left ventricular function. In the three other disease severity subgroups, composed of (2) normal ECG and abnormal LV function, (3) abnormal ECG and abnormal LV function without cardiac failure symptoms, and finally (4) abnormal ECG with abnormal LV function and symptoms of cardiac failure, the proportion of RV dilatation/global RVEF reduction/segmental wall motion abnormalities was, respectively, 55%/0%/64% for group (2), 79%/32%/74% for group (3), and 71%/100%/100% for group (4). Evaluating these results under the lens of modern knowledge about the complexity of cardiac imaging methods in assessing RV function, it is plausible to conclude that there is a progression of RV disease among patients with the various forms of CD, even if apparently no abnormalities were detected in the early asymptomatic phase of the disease with the methods employed. In contrast, Marin-Neto et al. [9], studying RV function with the more accurate and quantitative method of ECG-gated radionuclide angiocardiography, were able to demonstrate, in a carefully selected group of patients with the indeterminate form of disease, a reduction in RVEF despite the presence of preserved LVEF. This investigation rescued the concept of RV early functional impairment in patients with CD, in line with the pathological findings described above and the clinical manifestations already alluded here. This was possible due to the inherent fact that radionuclide angiography allowed the concomitant evaluation of both LV and RV. After the study of Carrasco et al., without known exceptions, all studies assessing the ventricular function with contrast ventriculography were focused exclusively on the LV and coronary angiography.

The results from the study with radionuclide angiography, coupled with previous anatomopathologic data already discussed, produced the hypothesis that RV myocardial damage may be an early and direct pathophysiological consequence of CC. This hypothesis also comprehends the notion that, in a scenario of low vascular pulmonary resistance (i.e., in the absence of LV systolic and diastolic dysfunction and its retrograde effects upon the pulmonary circulation), no clinical manifestations of the RV functional impairment would be detectable. However, within this setting of RV early impairment, when the left ventricle dysfunction occurs causing the rise in pulmonary vascular resistance, compensatory mechanisms of the RV achieve their limits. From this moment on, right-sided symptoms and signs may thus dominate the clinical expression of heart failure [34].

Mady et al., who had already shown right ventricle histologic abnormalities in 60% of patients with the indeterminate form of CD [36], also studied them, a year later, with invasive cardiac catheterization. Of note, the only hemodynamic abnormality seen in those patients with this form of CD was the RV end diastolic pressure elevation [47]. In the absence of any signs of LV dysfunction, it would be possible to conclude that the elevated diastolic pressure in the RV chamber might be due to the underlying structural abnormalities previously detected in the same patients.

Several years later, various studies using echocardiography as a noninvasive tool to assess RV geometry and function showed mixed results in diverse groups of patients with various forms of CD. Nunes et al., in 2004, using a nonquantitative echocardiography analysis, reported not being able to detect isolated RV dysfunction in a group of subjects in both early and later (28% of patients) stages of CC [48]. However, this conclusion was based only on a subjective global classification of RV systolic dysfunction. Moreover, in this study, only patients with some degree of left ventricle dilatation or systolic dysfunction were included, which, per se, makes the investigational task of finding isolated RV early involvement unfeasible. Furtado et al. also investigated the prevalence of RV systolic and diastolic dysfunction including its global performance index in a group of 60 subjects without evidence of cardiac disease by conventional diagnostic methods [49]. In contrast to the results of Nunes et al., the authors found a 26% prevalence of RV systolic dysfunction when using tissue Doppler systolic tricuspid velocity wave (S′). In that study no other echo parameters of RV function could differentiate the performance of patients with indeterminate form CD from normal controls. Ten years later, using* speckle tracking* as a new methodology echocardiography tool with potential to measure myocardium deformity, Barbosa et al. [50] reported that no RV longitudinal strain differences could be detected between patients with indeterminate form CD and normal controls.

Recently, Moreira et al. demonstrated, in a comparative investigation using cardiac magnetic resonance as a reference method, that all conventional echocardiographic parameters for right ventricular assessment studied, including TAPSE, S′ of tricuspid annulus, fractional area change, and right ventricular index of myocardial performance, have a very low sensitivity to detect right ventricular systolic dysfunction in patients with CD. In contrast, analysis of right ventricular deformation by using two-dimensional speckle tracking echocardiography yielded a high ability to differentiate patients with from those without RV systolic dysfunction. The right ventricular free wall strain had a remarkably higher sensitivity in comparison with traditional echocardiographic parameters to identify RV dysfunction [13].

Cardiac magnetic resonance methods have inherent advantages for the evaluation of RVEF, since volumes can be directly measured instead of being geometrically extrapolated [51]. Studying 158 subjects with CD using cardiac magnetic resonance, Moreira et al. described reduced right ventricular ejection fraction in 37% of the patients studied [51]. Although usually associated with LV dysfunction, isolated early right ventricular dysfunction was also found in a small subset (4.4%) of patients [51]. Furthermore, right ventricular dysfunction was independently associated with atrial fibrillation.

Based on all exposed data, we can conclude that detection of incipient right ventricle dysfunction is still dependent on technical evolution of cardiac diagnosis image tools. And this concept probably can be extrapolated to any disease with a potential to primarily involve the right ventricular myocardium.

The prognostic impact of RV dysfunction in CD is not yet completely explored. Nunes et al. evaluated 158 subjects, all with left ventricle dilatation and dysfunction during a follow-up of 34 ± 23 months. The RV dysfunction parameter evaluated, Tei index of ventricular performance, was a predictor of mortality [42]. These data can be taken as the first demonstration of the role of RV dysfunction as a possible prognostic marker in CC. Nevertheless, prognostic studies have still several barriers to transpose. One of them is to compare a new parameter with others previously established and this aspect was not correctly tested considering the validated Rassi score in CC [52, 53]. In contrast, although the myocardium performance index of right ventricle (Tei index) was already proven to be able to detect RV dysfunction in other scenarios [54], in CD this index could not identify RV involvement in the indeterminate form group of patients [55].

## 4. Heart Conduction Disturbances in Chagas Cardiomyopathy and Its Relation to RV Function

CD frequently affects the heart conduction system and the most frequently found conduction abnormality is right bundle branch block (RBBB), often associated with left anterior fascicle bundle branch block (LAFB) [56]. Prevalence of RBBB varies between* T. cruzi* chronically infected populations from 15% to 40% [17, 57]. Among 2756 patients enrolled in the BENEFIT trial, RBBB was found in roughly 52% and in 35% of cases it was associated with LAFBB. This association was also reproducible in experimental animal models of infection with the* T. cruzi* [19]. Histologically, these regions of the cardiac conduction system show chronic fibrosis and progressive obliteration [37].

It is not clear if RBBB is really a primarily manifestation or if it is secondary to myocardial damage extending to conduction system. Also, the role of RBBB in physiopathology of CD is not well understood. One previous study showed that, in a cohort of asymptomatic patients with RBBB, patients with CD have a worse prognostic regarding cardiac sudden death when compared to other patients with RBBB and no CD [58]. Although the influence of having CD was not compared with other classic prognostic factors, these data suggest the need for further studies looking specifically to the prognosis of patients with RBBB secondary to CD. Indeed, it is well documented that having a normal ECG is a marker of good prognosis in CD but the prognostic meaning of an isolated RBBB due to CD is unclear [59].

The real contribution of this electrical disturbance to RV dysfunction has not been investigated. This contrasts with the conspicuous evidence that left bundle branch block (LBBB) is associated with left ventricle dysfunction [60]. Although in other disease scenarios RBBB is usually an expression of RV pressure and volume overload, in CD this electrical disturbance could be a primary reflection of myocardial damage. The understanding about the role of RBBB in inducing delayed RV contraction in RV calculations of function by cardiac diagnosing methods is frequently ignored. This effect was tested using CMR by Marterer et al., concluding that ignoring the RV physiology in RBBB patients leads to a statistically significant underscoring of RV performance parameters [61]. Thus, the impact of RBBB in CD as a primary mechanism to RV mechanical dyssynchrony and dysfunction warrants further investigation with dedicated study design.

## 5. Current Imaging Evaluation of the Right Ventricle in CD: Focus on Noninvasive Cardiac Diagnostic Tools

### 5.1. Echocardiography

Evaluation of right ventricle anatomy and function with echocardiography is always challenging. Right ventricle unique macroscopic morphology, which includes an inflow and outflow regions and the main cavity, cannot be represented by a simple mathematical geometric model. Because of this, geometric extrapolations to estimate chamber volumes, so useful for bidimensional echocardiographic assessment of the LV, cannot be applied in the case of the RV [62]. Although understanding right ventricle involvement in several disease processes has gained a recent interest, it is still not yet adequately addressed in cardiology [63]. Tridimensional echocardiography, while promising, still has some difficulties when imaging the right ventricle, as the need to improve the technical spatial resolution to assess the right ventricle free wall [64]. Other unique characteristics of right ventricle are additional barriers, as the thin myocardium walls, its prominent trabeculation, and its contraction mechanics, characterized by a predominant long-axis (apex-to-basal) shortening of myocardium fibers.

**Figure 4:**
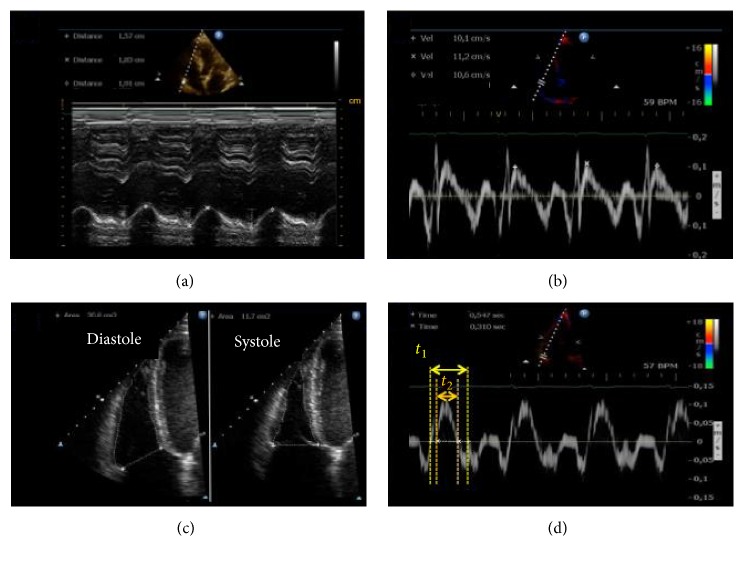
Echocardiographic conventional parameters of right ventricle (RV) evaluation. (a) Tricuspid Annular Peak Systolic Excursion (TAPSE) from M-mode. (b) Systolic wave velocity (*S*′) of the lateral portion of tricuspid annulus; (c) area measurements in diastole and systole from bidimensional images to calculate fractional area change (FAC); (d) time measurements from tissue Doppler curves in tricuspid annulus to calculate myocardial performance index (MPI). [13]. Adapted with permission from Elsevier, license number 4143761084119.

Recognizing these problems, a guide on how to measure right ventricle geometry and function with echocardiography was released in 2010 and was further updated and included in an echocardiography chamber quantification recommendation in 2015 [65, 66]. The document comprises bidimensional linear measures of RV cavity at different levels and projections and functional nonvolumetric parameters such as longitudinal excursion of tricuspid annulus (TAPSE), tissue Doppler velocity of systolic wave at the lateral portion of tricuspid annulus (S′ wave), fractional area change (FAC) during systole, myocardium performance index (MPI) also recognized as Tei index [67], and the estimate of *dP*/*dT* from systolic time of the tricuspid regurgitation Doppler spectrum [65] (Figure 4).

Considering the overall neglectful cardiology approach to the right ventricle, it is understandable why the same gaps of knowledge also exist for CD studies. Acquatella, in an outstanding publication survey in 2007, extensively reviewed echo contributions to the field of CD without fully addressing the mixed results of studies of echocardiographic evaluation of RV in CD [68]. Despite this neglect, from most echocardiographic studies primarily focusing on the LV assessment, it is possible to conclude that 2D echocardiography can detect right ventricular systolic dysfunction in a high proportion of patients who already have decreased global LV systolic impairment, even at earlier stages of the cardiomyopathy [13, 48]. Other publications also addressed the potential of echocardiographic conventional parameters such as *S*′, TAPSE, and MPI to detect right ventricular dysfunction in more advanced stages of disease and symptomatic patients [69]. Some publications also endorsed the ability of echocardiographic parameters to detect right ventricle dysfunction even in patients with the indeterminate form of the disease using tissue Doppler techniques [49, 70]. However, using myocardium performance index (MPI), investigators were not able to differentiate patients with the indeterminate form of CD from controls [49]. Also, using 2D speckle tracking, Barbosa et al., in a study of biventricular function, published negative results of strain evaluation to differentiate patients with indeterminate form of CD from controls [50].

**Figure 5:**
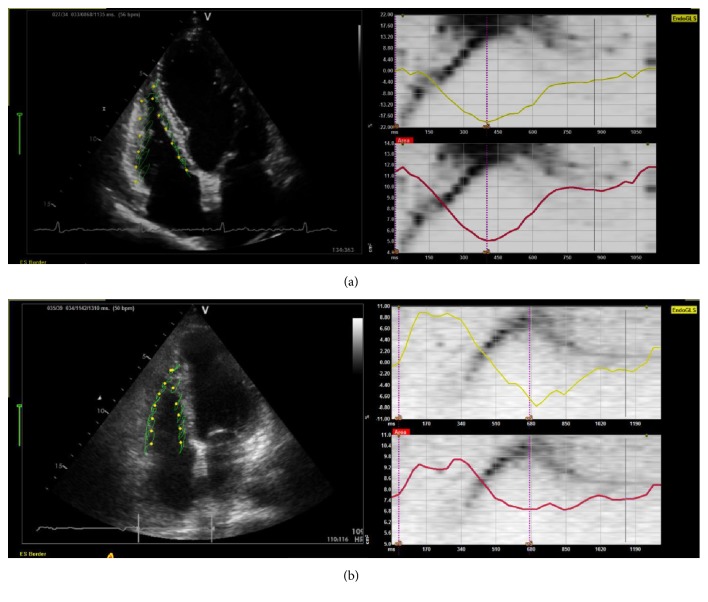
Myocardial deformation analysis of right ventricle (RV) from speckle tracking echocardiography. Strain analysis of RV from representative CC patients demonstrating preserved systolic function in Panel (a) (GLS: −17.8%, FAC: 55.7%) and reduced systolic function in Panel (b) (GLS: −8.4%, FAC: 10.5). Both patients have preserved left ventricle ejection fraction. Graphics in each panel represent myocardial strain in % (superior right, yellow trace) and RV area change in cm^2^ (inferior right, red trace). RV: right ventricle; EndoGLS: Endocardial Global Longitudinal Strain; FAC: Fractional Area Change; es: end-systolic time.

*Speckle tracking* is a relatively new echocardiography tool and discussion about its methodology advantage over other approaches is still a topic of interest [71]. With this technique, the myocardium systolic function can be measured along the entire myocardium as deformation (Figure 5). The myocardium deformation analysis is relatively independent from overload conditions [72, 73] and because of this, in theory, it can be applied to detect myocardial damage even when compensatory physiological adjustments are still preserving the ejection fraction within normal limits [72]. Normality values of RV strain by 2D speckle tracking analysis were recently published [74].

Most of published data using* speckle tracking* in CD were focused on left ventricle analysis assessment [50, 75–77]. These investigations used specific vendor software for the analysis and their methodological approach, mainly about timing of measured peak (if global or systolic), end-systole definitions, and also if endocardial or mesocardial layer, were neither technically uniform [50, 75] nor even were they completely reported in some of them. A definite standardization [71] in speckle tracking measurements is necessary to evolve the applicability of this method not only in the evaluation of LV deformation but also for the RV assessment. Results of a recent published study following a standardization methodology of measurements of speckle tracking concluded that right ventricular free wall strain measurement seems to be a method of choice [13], since the sensitivity of the conventional echocardiographic parameters to detect right ventricular dysfunction in CD is intrinsically very low.

In summary, echocardiography can be used to detect RV dysfunction in CD but with some inherent methodological limitations.* Speckle tracking* is a promising tool to assess RV dysfunction in this disease overcoming the lack of sensitivity of conventional 2D and Doppler measurements of function.

### 5.2. Cardiac Magnetic Resonance

**Figure 6:**
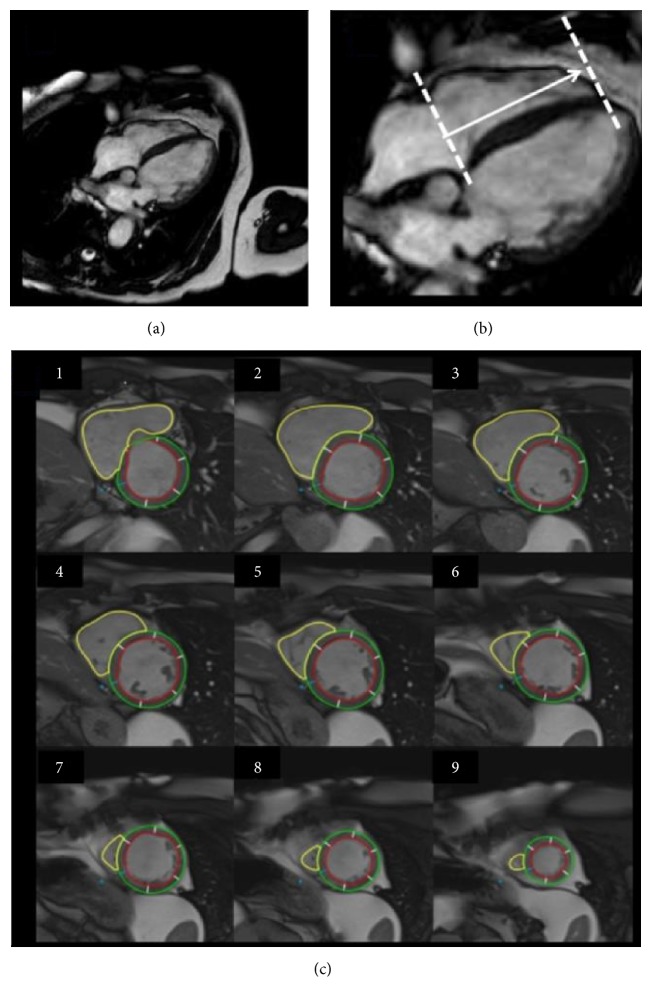
Volumetric assessment of the RV using CMR. Image acquired from cine-resonance using pulse sequences SSFP* (Steady-State Free Precession)*, showing the tomographic slicing of RV long-axis and limiting adjustments to transversal views from base to apex ((a) and (b)). Yellow lines shows delineation of endocardial borders of RV (c) to further calculation of volumes. Adapted from Moreira, HT (2015). “Análise ecocardiográfica do ventrículo direito na doença de Chagas: estudo comparativo com a ressonância magnética cardíaca” (Doctoral Dissertation), University of São Paulo, Ribeirão Preto, Brazil, Circ Cardiovasc Imaging, 2017, 10:e005571. Adapted with permission from Wolters Kluwer, license number 4143770060198.

**Figure 7:**
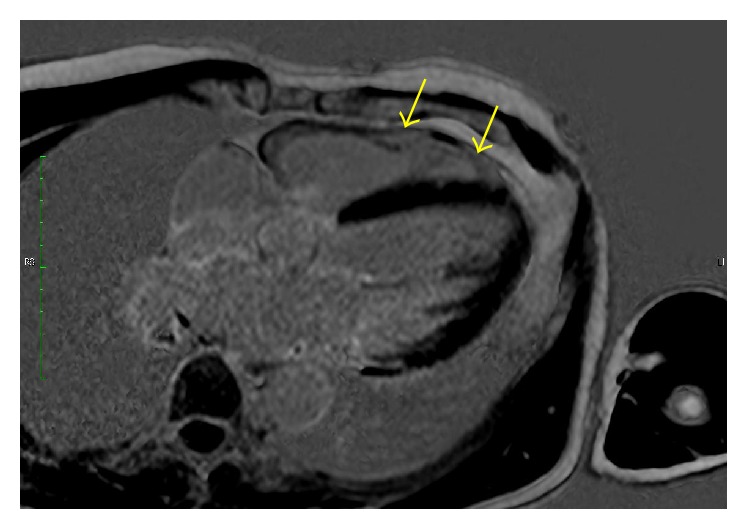
Four-chamber slice using CMR with LGE (late gadolinium enhancement) sequence in a patient with Chagas cardiomyopathy. Distinct extensive areas of fibrosis (white signal) (yellow arrows) are seen in the right ventricle free wall. Right ventricle dimensions are still within normal limits. Discrete isolated areas of fibrosis are also noted in the left ventricle.

With its inherent capability for the direct measurement of chamber volumes and the calculation of biventricular ejection fraction without geometric extrapolations, CMR is clearly a more advantageous method than echocardiography for that purpose. In this regard, the method is already considered a gold standard of ventricle volumes quantification [78]. This has been assumed in general, despite the fact that it has not been studied in comparison with the estimation of ejection fraction based on ECG-gated radionuclide angiography. In the absence of direct comparative studies of both methods, it may well be that the nuclear method, which also has no need for any geometric extrapolation (scintigraphy counts are directly proportional to volumes throughout the cardiac cycle), and has the advantage of allowing a precise averaging of hundreds of cardiac cycles, may stand the test of a comparison with CMR. In addition, the method of measuring EF with CMR has not been fully standardized, and there are still challenges on its way to more widespread clinical application. In particular, right ventricle ejection fraction normal reference values are still not consolidated. Despite these caveats, it is relevant to emphasize that in CD patients CMR can be used not only to calculate ventricular volumes and ejection fraction (Figure 6) but also to characterize myocardial tissue alterations (Figure 7).

The utility of CMR in CD was first demonstrated for the study of left ventricle systolic function and the detection of left ventricle myocardial fibrous tissue and its significance [79–82]. As mentioned above, a recent study by Moreira et al., using CMR, evaluated the prevalence of RV systolic dysfunction in a sizable population of patients with chronic CD. In that study a reduction of right ventricular ejection fraction was detected in 37% of the patients studied, usually associated with left ventricle dysfunction. Corroborating the findings from previous studies with other methods, Moreira et al. showed the presence of right ventricular systolic dysfunction in a small subset of these* T. cruzi* chronically infected subjects with preserved left ventricular ejection fraction [51].

Therefore, even considering the intrinsic difficulties in using CMR in clinical settings, most of them related to cost and availability, especially curtailed by scarce resources in endemic regions of CD, there is now a definite drive for the clinical approach with CMR to evaluate early biventricular damage in CD.

### 5.3. Future Perspectives

#### 5.3.1. Echocardiography

Recent research using a mammalian experimental model of* T. cruzi* infection that closely mimics human chronic Chagas, with the advantage of a relatively short 8–10 months of evolution to chronic phase, has allowed the integration of echocardiography analyses with anatomopathologic findings [83, 84]. Somewhat unexpectedly, it was first shown that left ventricle segmental motion abnormalities are more related to the underlying inflammation process than to myocardium fibrosis [85]. This is an emerging area of research with a great potential for “bench to bed” approach. Future correlation between anatomopathologic studies and clinical studies can advance knowledge of the disease pathophysiology and of therapeutic unfolding. Translational science thinking can guide clinical research to design studies testing a more detailed segmental systolic quantification not only in the left but also in the right ventricle to detect early myocardium involvement. Regional* speckle tracking* quantification can be a useful tool, despite its reproducibility still being a controversy theme.

#### 5.3.2. Cardiac Magnetic Resonance

Based on the concepts described along this review of CC being an immune-inflammatory disease affecting both the left and the right ventricles, CMR has the potential to detect not only fibrosis but also myocardium edema and inflammation, in order to further extend seeking for detection of early myocardial damage. CMR techniques as T2 sequences are recently emerging in diverse scenarios of myocardial diseases, including infarction and acute myocarditis [86], and the utility of the method in CD is an open field for new exploits [80]. The potential role of the method in allowing a more accurate assessment of the right ventricular function has already been documented in various clinical settings [87].

## 6. Conclusion

Right ventricle role in cardiovascular physiology and pathology has been historically neglected. Cardiology science is still “on the way” to understand the right ventricle role into cardiovascular physiology and to detect structural and function abnormalities in this geometrically complex chamber.

Different from other dilated cardiomyopathies, CD has a unique characteristic of frequently early and progressive damage involving cell death, inflammation, and fibrosis not only in the left but also in the right ventricle myocardium. Evolution of cardiac imaging modalities added current analyses of myocardial deformation by speckle tracking echocardiography and both chamber volumetric evaluation and tissue characterization using cardiac magnetic resonance. These novel technologies offer new perspective to study right ventricular involvement and to understand the associated aspects of CD progression in terms of malignant arrhythmias and heart failure. Sudden death, the most frequent mode of death in patients with CC, occurring even when there is preserved global LV systolic function, should be especially amenable to investigations using CMR.

## Additional Points

*Methods for Search Strategy and Selections*. Data for this review were identified by searching Medline, PubMed, and Lilacs Databases and publications in English, Spanish, or Portuguese language were accepted. No other kind of selection of manuscripts to be cited was applied, considering most of available published data.

## References

[1] Weber K. T., Janicki J. S., Shroff S., Fishman A. P. (1981). Contractile mechanics and interaction of the right and left ventricles. *The American Journal of Cardiology*.

[2] Janicki J. S., Weber K. T. (1980). Factors influencing the diastolic pressure-volume relation of the cardiac ventricles. *Federation Proceedings*.

[3] Starr I., Jeffers W. A., Meade R. H. (1943). The absence of conspicuous increments of venous pressure after severe damage to the right ventricle of the dog, with a discussion of the relation between clinical congestive failure and heart disease. *American Heart Journal*.

[4] Brooks H., Holland R., Al-Sadir J. (1977). Right ventricular performance during ischemia: an anatomic and hemodynamic analysis. *The American journal of physiology*.

[5] Dell'Italia L. J. (2012). Anatomy and Physiology of the Right Ventricle. *Cardiology Clinics*.

[6] Basso C., Corrado D., Marcus F. I., Nava A., Thiene G. (2009). Arrhythmogenic right ventricular cardiomyopathy. *The Lancet*.

[7] Rassi A., Rassi A., Marin-Neto J. A. (2010). Chagas disease. *The Lancet*.

[8] A. Z. Prata A., Guimarães A., H. M. e. a. Shaper A. Chagas Heart Disease.

[9] Marin-Neto J. A., Marzullo P., Sousa A. C. (1988). Radionuclide angiographic evidence for early predominant right ventricular involvement in patients with Chagas' disease. *Canadian Journal of Cardiology*.

[10] Moreira H. T., Volpe G. J., Trad H. S. (2014). Right ventricular dysfunction in Chagas'cardiomyopathy. Primary involvement or a resonant manifestation of left ventricular dysfunction?. *Journal of Cardiovascular Magnetic Resonance*.

[11] Prado C. M., Celes M. R. N., Malvestio L. M. (2012). Early dystrophin disruption in the pathogenesis of experimental chronic Chagas cardiomyopathy. *Microbes and Infection*.

[12] Rossi M. A. (1991). The pattern of myocardial fibrosis in chronic Chagas' heart disease. *International Journal of Cardiology*.

[13] Moreira H. T., Volpe G. J., Marin-Neto J. A. (2017). Right ventricular systolic dysfunction in chagas disease defined by speckle-tracking echocardiography: a comparative study with cardiac magnetic resonance imaging. *Journal of the American Society of Echocardiography*.

[14] WHO Expert Committee on the Control of Chagas Disease (1991). Control of Chagas' disease: second report of the WHO expert committee [meeting held in Buenos Aires from 16 to 20 October 1989]. *WHO Tecnical Report Series*.

[15] Global Burden of Disease Study C. (2015). Global, regional, and national incidence, prevalence, and years lived with disability for 301 acute and chronic diseases and injuries in 188 countries, 1990-2013: a systematic analysis for the Global Burden of Disease Study 2013. *The Lancet*.

[16] Bern C., Kjos S., Yabsley M. J., Montgomery S. P. (2011). *Trypanosoma cruzi* and Chagas' disease in the united states. *Clinical Microbiology Reviews*.

[17] Traina M. I., Hernandez S., Sanchez D. R. (2017). Prevalence of Chagas Disease in a U.S. Population of Latin American Immigrants with Conduction Abnormalities on Electrocardiogram. *PLOS Neglected Tropical Diseases*.

[18] Bonney K. M., Engman D. M. (2008). Chagas heart disease pathogenesis: One mechanism or many?. *Current Molecular Medicine*.

[19] Andrade Z. A., Andrade S. G., Oliveria G. B., Alonso D. R. (1978). Histopathology of the conducting tissue of the heart in Chagas' myocarditis. *American Heart Journal*.

[20] Amorin D. S., Mello de Oliveira J. A., Meira de Oliveira J. S. (1976). Heart block in Chagas' disease. *Arquivos Brasileiros de Cardiologia*.

[21] Marin-Neto J. A., Bromberg-Marin G., Pazin-Filho A., Simões M. V., Maciel B. C. (1998). Cardiac autonomic impairment and early myocardial damage involving the right ventricle are independent phenomena in Chagas' disease. *International Journal of Cardiology*.

[22] Köberle F. (1968). Chagas' Disease and Chagas' Syndromes: The Pathology of American Trypanosomiasis. *Advances in Parasitology*.

[23] Marin-Neto J. A., Cunha-Neto E., Maciel B. C., Simões M. V. (2007). Pathogenesis of chronic Chagas heart disease. *Circulation*.

[24] Rossi M. A., Tanowitz H. B., Malvestio L. M. (2010). Coronary microvascular disease in chronic chagas cardiomyopathy including an overview on history, pathology, and other proposed pathogenic mechanisms. *PLoS Neglected Tropical Diseases*.

[25] Simões M. V., Pintya A. O., Bromberg-Marin G. (2000). Relation of regional sympathetic denervation and myocardial perfusion disturbance to wall motion impairment in Chagas' cardiomyopathy. *American Journal of Cardiology*.

[26] Morris S. A., Tanowitz H. B., Wittner M., Bilezikian J. P. (1990). Pathophysiological insights into the cardiomyopathy of Chagas' disease. *Circulation*.

[27] Andrade D. V., Gollob K. J., Dutra W. O. (2014). Acute Chagas Disease: New Global Challenges for an Old Neglected Disease. *PLoS Neglected Tropical Diseases*.

[28] Nóbrega A. A., Garcia M. H., Tatto E. (2009). Oral transmission of chagas disease by consumption of Açaí palm fruit, Brazil. *Emerging Infectious Diseases*.

[29] Tavora M. Z., Mehta N., Silva R. M., Gondim F. A., Hara V. M., de Paola A. A. (1999). Characteristics and identification of sites of chagasic ventricular tachycardia by endocardial mapping. *Arquivos Brasileiros de Cardiologia*.

[30] Andrade S. G., Sadigursky M. (1987). The conduction system of the heart in mice chronically infected with Trypanosoma cruzi: histopathological lesions and electrocardiographic correlations. *Memorias do Instituto Oswaldo Cruz*.

[31] Koberle F. (1957). The chronic Chagas cardiopathy. *Virchows Archiv Fur Pathologische Anatomie Und Physiologie Und Fur Klinische Medizin*.

[32] Rassi A., Rassi A., Marcondes de Rezende J. (2012). American trypanosomiasis (Chagas disease). *Infectious Disease Clinics of North America*.

[33] Andrade J. P., Marin-Neto J. A., Paola A. A. (2011). I Latin American guidelines for the diagnosis and treatment of Chagas cardiomyopathy. *Arquivos Brasileiros de Cardiologia*.

[34] Marin-Neto J. A., Andrade Z. A. (1991). Why is there predominance of right heart failure in Chagas' disease?. *Arquivos Brasileiros de Cardiologia*.

[35] Barreto A. C., Higuchi Mde L., Da Luz P. L. (1989). Comparison of histologic changes in Chagas'cardiomyopathy and dilated cardiomyopathy. *Arquivos Brasileiros de Cardiologia*.

[36] Mady C., Barretto A. C., Stolf N. (1981). Endomyocardial biopsy in the indeterminate form of Chagas' disease. *Arquivos Brasileiros de Cardiologia*.

[37] Hagar J. M., Rahimtoola S. H. (1995). Chagas' heart disease. *Current Problems in Cardiology*.

[38] Higuchi M. d. L., Lopes E. A., Barretto A. C. (1985). Endomyocardial biopsy of the right ventricle. Significance of the ultrastructural changes in Chagas' cardiopathy. *Arquivos Brasileiros de Cardiologia*.

[39] Barretto A. C. P., Mady C., Arteage-Fernandez E. (1986). Right ventricular endomyocardial biopsy in chronic Chagas' disease. *American Heart Journal*.

[40] Lescure F.-X., Le Loup G., Freilij H. (2010). Chagas disease: Changes in knowledge and management. *The Lancet Infectious Diseases*.

[41] Freitas H. F. G., Chizzola P. R., Paes Â. T., Lima A. C. P., Mansur A. J. (2005). Risk stratification in a Brazilian hospital-based cohort of 1220 outpatients with heart failure: Role of Chagas' heart disease. *International Journal of Cardiology*.

[42] Nunes M. D. C. P., Rocha M. O. C., Ribeiro A. L. P. (2008). Right ventricular dysfunction is an independent predictor of survival in patients with dilated chronic Chagas' cardiomyopathy. *International Journal of Cardiology*.

[43] Simoes M. V., Nonino A., Simoes B. P., Almeida-Filho O. C., Maciel B. C., Marin-Neto J. A. (1994). Reagudization of Chagas myocarditis inducing exclusive right ventricular failure. *Arquivos Brasileiros de Cardiologia*.

[44] Simões M. V., Soares F. A., Marin-Neto J. (1995). Severe myocarditis and esophagitis during reversible long standing Chagas' disease recrudescence in immunocompromised host. *International Journal of Cardiology*.

[45] Souza F. F., Castro-e-Silva O., Marin Neto J. A. (2008). Acute Chagasic Myocardiopathy After Orthotopic Liver Transplantation With Donor and Recipient Serologically Negative for Trypanosoma cruzi: A Case Report. *Transplantation Proceedings*.

[46] Carrasco H. A., Medina M., Inglessis G., Fuenmayor A., Molina C., Davila D. (1983). Right ventricular function in Chagas disease. *International Journal of Cardiology*.

[47] Mady C., de Moraes A. V., Galiano N., Decourt L. V. (1982). Hemodynamic study of the indeterminate form of Chagas' disease. *Arquivos Brasileiros de Cardiologia*.

[48] Nunes M. D. C. P., Barbosa M. D. M., Brum V. A. A., Rocha M. O. D. C. (2004). Morphofunctional characteristics of the right ventricle in Chagas' dilated cardiomyopathy. *International Journal of Cardiology*.

[49] Furtado R. G., Do Carmo Rassi Frota D., Silva J. B. M. (2015). Right ventricular doppler echocardiographic study of indeterminate form of chagas disease. *Arquivos Brasileiros de Cardiologia*.

[50] Barbosa M. M., Rocha M. O. C., Vidigal D. F. (2014). Early detection of left ventricular contractility abnormalities by two-dimensional speckle tracking strain in Chagas' disease. *Echocardiography*.

[51] Moreira H. T., Volpe G. J., Marin-Neto J. A. (2017). Evaluation of Right Ventricular Systolic Function in Chagas Disease Using Cardiac Magnetic Resonance Imaging. *Circulation: Cardiovascular Imaging*.

[52] Rassi A., Rassi A., Little W. C. (2006). Development and validation of a risk score for predicting death in Chagas' heart disease. *New England Journal of Medicine*.

[53] Rassi A., Rassi A. (2010). Predicting prognosis in patients with Chagas disease: why are the results of various studies so different?. *International Journal of Cardiology*.

[54] Pavlicek M., Wahl A., Rutz T. (2011). Right ventricular systolic function assessment: Rank of echocardiographic methods vs. cardiac magnetic resonance imaging. *European Journal of Echocardiography*.

[55] Pazin-Filho A., Romano M. M. D., Gomes Furtado R. (2007). Left Ventricular Global Performance and Diastolic Function in Indeterminate and Cardiac Forms of Chagas' Disease. *Journal of the American Society of Echocardiography*.

[56] Cardoso R., Garcia D., Fernandes G. (2016). The Prevalence of Atrial Fibrillation and Conduction Abnormalities in Chagas' Disease: A Meta-Analysis. *Journal of Cardiovascular Electrophysiology*.

[57] Williams-Blangero S., Magalhaes T., Rainwater E., Blangero J., Correa-Oliveira R., VandeBerg J. L. (2007). Electrocardiographic characteristics in a population with high rates of seropositivity for Trypanosoma cruzi infection. *American Journal of Tropical Medicine and Hygiene*.

[58] Pimenta J., Valente N., Miranda M. (1999). Long-term follow up of asymptomatic chagasic individuals with intraventricular conduction disturbances, correlating with non-chagasic patients. *Revista da Sociedade Brasileira de Medicina Tropical*.

[59] Maguire J. H., Hoff R., Sherlock I. (1987). Cardiac morbidity and mortality due to Chagas' disease: Prospective electrocardiographic study of a Brazilian community. *Circulation*.

[60] Vernooy K., Verbeek X. A. A. M., Peschar M., Prinzen F. W. (2003). Relation between Abnormal Ventricular Impulse Conduction and Heart Failure. *Journal of Interventional Cardiology*.

[61] Marterer R., Hongchun Z., Tschauner S., Koestenberger M., Sorantin E. (2015). Cardiac MRI assessment of right ventricular function: impact of right bundle branch block on the evaluation of cardiac performance parameters. *European Radiology*.

[62] Sheehan F., Redington A. (2008). The right ventricle: Anatomy, physiology and clinical imaging. *Heart*.

[63] Haddad F., Hunt S. A., Rosenthal D. N., Murphy D. J. (2008). Right ventricular function in cardiovascular disease, part I: anatomy, physiology, aging, and functional assessment of the right ventricle. *Circulation*.

[64] Lang R. M., Badano L. P., Tsang W. (2012). EAE/ASE recommendations for image acquisition and display using three-dimensional echocardiography. *European Heart Journal Cardiovascular Imaging*.

[65] Rudski L. G., Lai W. W., Afilalo J. (2010). Guidelines for the echocardiographic assessment of the right heart in adults: a report from the American Society of Echocardiography endorsed by the European Association of Echocardiography, a registered branch of the European Society of Cardiology, and the Canadian Society of Echocardiography. *Journal of the American Society of Echocardiography*.

[66] Lang R. M., Badano L. P., Mor-Avi V. (2015). Recommendations for cardiac chamber quantification by echocardiography in adults: an update from the American Society of Echocardiography and the European Association of Cardiovascular Imaging. *European Heart Journal—Cardiovascular Imaging*.

[67] Tei C., Ling L. H., Hodge D. O. (1995). New index of combined systolic and diastolic myocardial performance: a simple and reproducible measure of cardiac function—a study in normals and dilated cardiomyopathy. *Journal of Cardiology*.

[68] Acquatella H. (2007). Echocardiography in Chagas heart disease. *Circulation*.

[69] Nascimento C. A. S., Gomes V. A. M., Silva S. K. (2013). Left atrial and left ventricular diastolic function in chronic chagas disease. *Journal of the American Society of Echocardiography*.

[70] Barros M. V. L., Machado F. S., Ribeiro A. L. P., Da Costa Rocha M. O. (2002). Detection of early right ventricular dysfunction in Chagas' disease using Doppler tissue imaging. *Journal of the American Society of Echocardiography*.

[71] Voigt J.-U., Pedrizzetti G., Lysyansky P. (2015). Definitions for a common standard for 2D speckle tracking echocardiography: Consensus document of the EACVI/ASE/industry task force to standardize deformation imaging. *Journal of the American Society of Echocardiography*.

[72] Blessberger H., Binder T. (2010). Two dimensional speckle tracking echocardiography: Clinical applications. *Heart*.

[73] Blessberger H., Binder T. (2010). Two dimensional speckle tracking echocardiography: Basic principles. *Heart*.

[74] Muraru D., Onciul S., Peluso D. (2016). Sex- and method-specific reference values for right ventricular strain by 2-dimensional speckle-tracking echocardiography. *Circulation: Cardiovascular Imaging*.

[75] Gomes V. A. M., Alves G. F., Hadlich M. (2016). Analysis of Regional Left Ventricular Strain in Patients with Chagas Disease and Normal Left Ventricular Systolic Function. *Journal of the American Society of Echocardiography*.

[76] García-Álvarez A., Sitges M., Regueiro A. (2011). Myocardial deformation analysis in chagas heart disease with the use of speckle tracking echocardiography. *Journal of Cardiac Failure*.

[77] Lima M. S. M., Villarraga H. R., Abduch M. C. D. (2016). Comprehensive left ventricular mechanics analysis by speckle tracking echocardiography in Chagas disease. *Cardiovascular Ultrasound*.

[78] Hundley W. G., Bluemke D. A., Finn J. P. (2010). ACCF/ACR/AHA/NASCI/SCMR 2010 expert consensus document on cardiovascular magnetic resonance: a report of the American College of Cardiology Foundation Task Force on Expert Consensus Documents. *Journal of the American College of Cardiology*.

[79] Rochitte C. E., Oliveira P. F., Andrade J. M. (2005). Myocardial delayed enhancement by magnetic resonance imaging in patients with Chagas' disease: A marker of disease severity. *Journal of the American College of Cardiology*.

[80] Torreão J. A., Ianni B. M., Mady C. (2015). Myocardial tissue characterization in Chagas' heart disease by cardiovascular magnetic resonance. *Journal of Cardiovascular Magnetic Resonance*.

[81] Kalil R., Bocchi E. A., Ferreira B. M. (1995). Magnetic resonance imaging in chronic Chagas cardiopathy. Correlation with endomyocardial biopsy findings. *Arquivos brasileiros de cardiologia*.

[82] de Mello R. P., Szarf G., Schvartzman P. R. (2012). Delayed enhancement cardiac magnetic resonance imaging can identify the risk for ventricular tachycardia in chronic Chagas' heart disease. *Arquivos Brasileiros de Cardiologia*.

[83] Bilate A. M. B., Salemi V. M. C., Ramires F. J. A. (2003). The Syrian hamster as a model for the dilated cardiomyopathy of Chagas' disease: a quantitative echocardiographical and histopathological analysis. *Microbes and Infection*.

[84] Ramirez L. E., Lages-Silva E., Soares J. M., Chapadeiro E. (1994). The hamster (Mesocricetus auratus) as experimental model in Chagas' disease: parasitological and histopathological studies in acute and chronic phases of Trypanosoma cruzi infection. *Revista da Sociedade Brasileira de Medicina Tropical*.

[85] De Oliveira L. F. L., Romano M. M. D., De Carvalho E. E. V. (2016). Histopathological correlates of global and segmental left ventricular systolic dysfunction in experimental chronic chagas cardiomyopathy. *Journal of the American Heart Association*.

[86] Amano Y., Tachi M., Tani H., Mizuno K., Kobayashi Y., Kumita S. (2012). T2-weighted cardiac magnetic resonance imaging of edema in myocardial diseases. *The Scientific World Journal*.

[87] Schmidt A., Maciel B. C., Marin-Neto J. A. (2011). Clinical Trial Report: Time is Resonant for Right Ventricular Evaluation. *Current Cardiovascular Imaging Reports*.

